# Mistletoe Berry Outline Mapping with a Path Curve Function and Recording the Circadian Rhythm of Their Phenotypic Shape Change

**DOI:** 10.3389/fpls.2016.01749

**Published:** 2016-11-25

**Authors:** Renatus Derbidge, Stephan Baumgartner, Peter Heusser

**Affiliations:** ^1^Institute of Integrative Medicine, University of Witten/HerdeckeWitten, Germany; ^2^Research Institute at the Goetheanum, Science SectionDornach, Switzerland; ^3^Hiscia Institute, Society for Cancer ResearchArlesheim, Switzerland

**Keywords:** mistletoe, plant movement, circadian rhythm, change of shape, shape mapping, form and function, phenotypic study

## Abstract

This paper presents a discovery: the change of the outline shape of mistletoe (*Viscum album* ssp. *album*) berries *in vivo* and *in situ* during ripening. It was found that a plant organ that is usually considered to merely increase in size actually changes shape in a specific rhythmic fashion. We introduce a new approach to chronobiological research on a macro-phenotypic scale to trace changes over long periods of time (with a resolution from hours to months) by using a dynamic form-determining parameter called Lambda (λ). λ is known in projective geometry as a measure for pertinent features of the outline shapes of egg-like forms, so called path curves. Ascertained circadian changes of form were analyzed for their correlation with environmental factors such as light, temperature, and other weather influences. Certain weather conditions such as sky cover, i.e., sunshine minutes per hour, have an impact on the amplitude of the daily change in form. The present paper suggests a possible supplement to established methods in chronobiology, as in this case the dynamic of form-change becomes a measurable feature, displaying a convincing accordance between mathematical rule and plant shape.

## Introduction

In the present research project we were interested in analyzing the form or shape of a plant organ *in vivo* and *in situ* and recording how it changes over time. The main focus in recent biology is on phenomena on a cellular and genetic level. To our thinking the link between biological forms on a phenotypic level with chronobiological findings deserves more attention, since form and shapes of biological organs are such apparent and pertinent features. We applied mathematical functions to the outline shape of mistletoe berries (*Viscum album* ssp. *album* L.). These so-called “path curves” accord accurately with the shape of mistletoe berries (Flückiger and Baumgartner, 2003; Baumgartner et al., 2004). Furthermore, path curves are suited to revealing even small phenotypic changes and rhythms of form. A parameter called Lambda (λ) defines the outline shape of path curves (Edwards, 2003; Klein, 2006, 2013; Derbidge et al., 2013). λ allows exact determination of the form derived from the profiles of the plant organs in question.

Our work is related to the research of the Scottish mathematician Edwards (1912–2004). For decades he measured the form of plant leaf buds, in particular those of trees during dormancy. He observed a fortnightly change of shape in almost all species, discovering that winter dormancy is not a static state, but that it also exhibits rhythmical phenomena (Clopper, 1994; Edwards, 2006). We refined his method to meet the needs and standards of current science by developing corresponding software (Derbidge et al., 2013).

In a previous publication we described the mathematical background of the path curves applied for fitting berry outlines, the software developed and the technical intra- and interrater variability to determine the shape-defining parameter λ (Derbidge et al., 2013). The present publication focuses on the non-mathematical methods applied in this study (mostly technical procedures for outdoor photography) and furthermore presents detailed results of four independent data sets (from four consecutive years) to identify any circadian rhythms present and to investigate the biological variability over 4 years. In this context, a possible modulation by weather effects is analyzed. The detected circadian rhythm seems to be independent from temperature but triggered by the condition of sky cloudiness, i.e., blue sky or cloud cover.

Developing a method to trace phenotypic changes in outline shape introduces a new approach to chronobiology, whereby rhythms in morphological change can be detected. In this way, a salient biological feature can be mapped in a non-invasively phenotypic way.

## Materials and Methods

In separate sections below, we describe the sequence of steps involved in the study: observations of the living, untouched berries still attached to their branches, digital photography, the artificial lighting situation for day and night photography, computerized image analysis to reveal the state of form (determination of the λ-value) and statistical analysis and correlations of λ with weather data. A separate section briefly explains what λ is and how it is used. The methodology combines two areas usually kept distinct: outdoor observations and (indoor) laboratory precision. The objects studied grow in a natural habitat in the open. Little huts around the area of interest shield a small space for “laboratory-like” conditions. The balance established here between usually separate approaches accords with the need to observe the plants in nature, but may also be problematic, as will be discussed.

### Mistletoe Biology

European mistletoe (*Viscum album* ssp. *album* L.) is an indeciduous, perennial, dioecious, half parasitic dicotyledonous plant growing on varied trees mainly in middle Europe. After 4–7 years (depending on the host tree and vitality of the mistletoe bush) it starts to flower. Flowering season is in late winter (February/March). After pollination (mainly by flies) a green berry develops. Inside the berry the new plant develops as an embryo without a hard shell or the usual dormancy period associated with seed producing plants. The full ripening time requires almost a year. This is congruent with the generally slow growth of mistletoe. In autumn, around September or October, the full size is reached (average of 9 mm in height and 8 mm in width, having a shape of a very round egg). It then turns white and transparent, so the green and photosynthetically active embryo can just barely be seen through the outer skin of the berry. According to unpublished data (Urech, personal communication) mistletoe berries gain weight until the end of December. Unpublished observations from the current study show that rhythmic fluctuations of form finishes in December or January, also corresponding to the time of year when the berries are fully ripe. The berries then remain on the plant until they are removed by external influences like extreme weather conditions or being eaten by birds. Berries can be found on the bush until summer of the following year. Quite often they are still in good shape, but clearly they gradually begin to wilt after December, indicating that the tension of the skin is decreasing (see Sallé, 1983; Büssing, 2000 for whole section).

### Mistletoe and Host Trees

We chose bushes of European mistletoe (*Viscum album* ssp. *album* L.) of at least 5–6 years in age, these being mature enough to develop berries. Since mistletoe grows on trees we chose bushes on lower branches that were in reach for easy handling. In a natural habitat this rarely happens. At the mistletoe research institute Hiscia in Arlesheim, Switzerland, mistletoe is artificially seeded on various host trees and also on lower branches. After testing the reaction of the mistletoe plants to growing on different host trees such as apple (*Malus domestica*), lilac (*Syringa vulgaris*), oak (*Quercus robur*), and pine (*Pinus sylvestris*) under surveillance in rain-protected conditions, we decided to concentrate on the oak host tree since the altered, semi-artificial observation environment (huts during observation period built around them with greenhouse effects) did neither affect mistletoe nor the host tree. In contrast fungal infections developed on mistletoe growing on apple and on the apple tree itself. Mistletoe growing on oaks (along with the oak branch itself) showed a resistant and robust behavior, i.e., the berries were stable in their ripening until the end of the observation period.

### Mistletoe and Huts

**FIGURE 1:**
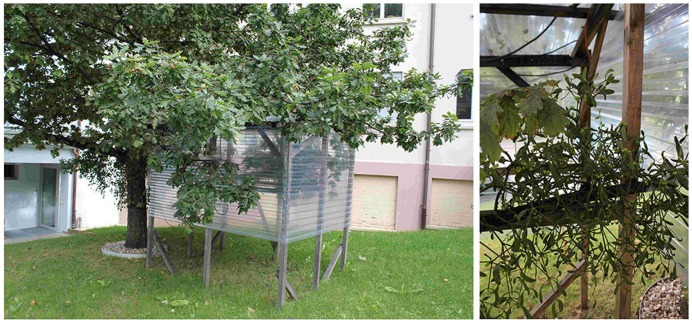
**A mistletoe hut. (Left)** The cabin shelters a branch with a bush of mistletoe on an oak tree (*Quercus robur*), approximately 35 years old. **(Right)** An isolated and sheltered mistletoe bush without any additional technical equipment.

In order to protect the digital cameras and the control equipment for regular photographing, huts were built around bushes of mistletoe growing on low branches of oak trees in the gardens of the Hiscia Institute in Arlesheim, Switzerland. Chosen for reasons of easy handling, the branches with the mistletoe bushes were positioned 1–2 m above the ground (**Figure 1**). The huts were constructed from untreated wood and corrugated PVC sheets that keep out rain and protect bush and equipment from wind. Since the PVC sheets are transparent, light conditions were not substantially altered. Roofs and sides were constructed so as to make air circulation possible. At least one side (pointing away from the main wind direction) and large openings above the ground and below the roof allow air to circulate and warm air to escape.

The observations start with the onset of berry ripening in September and are completed with the end of berry maturation in December. Thus, from January until September – when no observations take place – roofs and sides are removed. Otherwise, insects and fungi find favorable conditions that cause harm to the mistletoe plants due to the artificial situation, especially during summer with elevated temperature due to the greenhouse effect of the hut.

### The Camera-Support Construction and the Fixed Position of Mistletoe Berries

**FIGURE 2:**
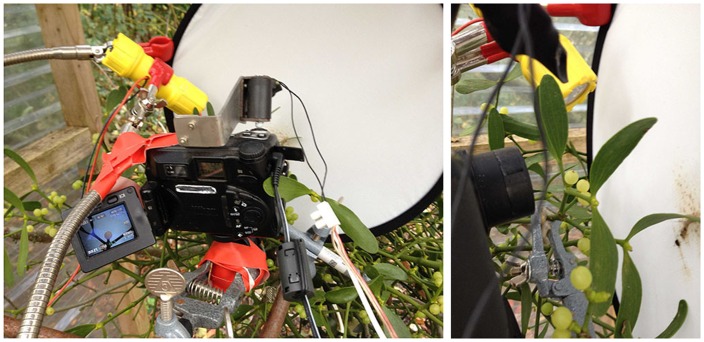
**Cabin and camera in use.** The hut (**Figure 1**) shelters the technical equipment as well as the berries to achieve a stable situation for the period of measurements (September to December). **(Left)** The camera with externally programmed electro-mechanical “finger” on the camera’s trigger; the LED flashlight (yellow) that switches on whenever a picture is taken. Lamp, camera and light reflection screen are attached and held by a solid metal structure with clamps and flexible but stable metal arms. The power cables are needed to run the camera, the “finger” (trigger) and the flashlight. **(Right)** A mistletoe berry between lens and light reflector screen.

The huts’ robust wooden structure allows a construction of metal rods and clamps used in chemical labs to maintain a flexible yet solid position to which the necessary camera and equipment can be attached. Mistletoe branches are also held in a fixed position. Thus, the camera can be adjusted at an exact angle of 90° to the main axis of the berry and at a distance of 3 cm from lens to the berry’s axis. Camera, lights and technical devices are shown in **Figure 2**.

### The Camera

We use Nikon Coolpix E5000 cameras, which have a large set of features, most importantly a close-up focus of up to 2 cm from lens to object. The flexible LCD-monitor allows excellent visibility control of the picture. All the features are separately and manually programmable. Triggerable electro-mechanical shutter release “fingers” (see Automatic Photography) allowed photographing in programmed intervals (see **Figure 2**).

The camera settings used were as follows:

– No flash
– Fixed aperture setting: shutter speed of 1/250 or 1/500 s (ensuring that the background, i.e., the light dispenser screen was always lighter than the focused object); focal length of 63 mm, focused at 3 cm
– 2560 × 1920 Pixels, average size between 800 and 1200 kilobytes
– Pictures saved as JPEG

All the above settings, as well as the time and date of the picture taken were saved in the JPEGs exif data.

### The Lighting

We use 9 Volt LED flashlights for lighting (see **Figure 2**). LEDs are a good alternative to other light sources (Jeong et al., 2012). They produce almost no warmth and only moderate electric and magnetic fields. To reduce influences as much as possible, the flashlight is switched on for only 3 s during photography. The light spectrum for LEDs shows a maximum in blue light wavelength but has an equal distribution (all visible colors in its spectrum) as measured by spectroscope (Carl Zeiss, Hand Spectroscope Nr. 9931).

**FIGURE 3:**
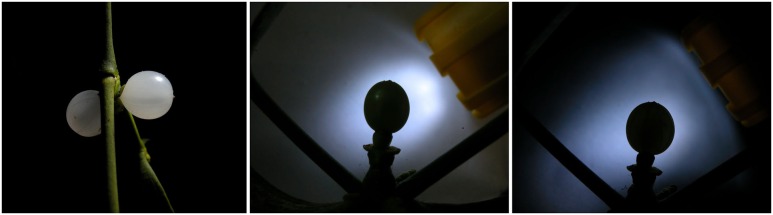
**The lighting situation. (Left)** Direct lighting produces somewhat blurry contrasts and unequal reflections. The indirect, passive lighting via a reflector screen produces “black” berries with equally lit (i.e., shaded) edges at night **(middle)** and daytime **(right)**. The LED flashlight is pointed at a light reflector. The indirect lighting results in comparable pictures both during the night and day.

To get equal light situations during day and night and to achieve optimal contrast for a clearly defined berry outline, the lighting was passive, i.e., via a reflector (**Figures 2** and **3**). Short exposure time (1/250 or 1/500 s) combined with flashlight reflection gave black silhouettes of the berry at any time of day (**Figure 3**).

### Automatic Photography

**FIGURE 4:**
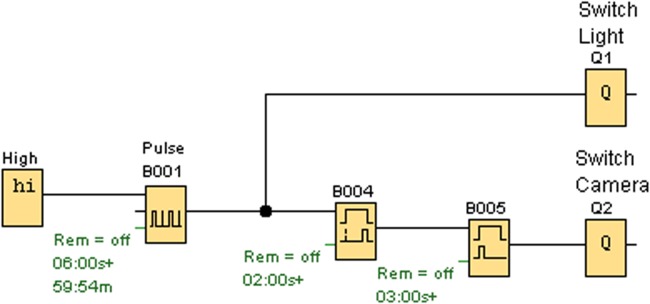
**The programmed scheme of the relay showing how lighting and the camera trigger are controlled (Q1 = Lighting, Q2 = Trigger)**.

We use a programmable relay (Logo! 230Rco by Siemens). This triggers the lighting by switching the flashlight on for 3 s and, with a delay of 1 s, activates the shutter release (“finger”) of the camera, causing the camera to take a picture (in a lit situation). The scheme of programming is shown in **Figure 4**.

### Measurements

The pictures are imported in the “LambdaFit” software, which is programmed to semi-automatically fit the outline shape of the object with its specific path curve situation (Derbidge et al., 2013). The software determines the contour of the shape and calculates a path curve starting from a set of predefined parameters (top and bottom of the object, and λ). The software further calculates the difference between the path curve and that of the outline shape, and minimizes this difference by iteration, varying the free parameters λ, width, length and the angle of the symmetry axis according to a defined mathematical procedure. The result of one such process is called a measurement, yielding data values for λ, and also width and length of the object. Due to the multi-parameter fit procedure, results of such measurements may vary slightly depending on the starting values of the curve fitting process. Due to this fact, multiple measurements are performed on one photograph in order to get stable mean fitting parameters and to determine variability of the fitting procedure (see Data Sets and Lambda Values). To avoid systematic errors randomized sets of pictures were subjected to measurements [e.g., sets of 144 photographs (6 days × 24 pictures); each picture measured 10 times]. The outline shape described by λ is independent of the size of the photographed object and of the distance between lens and object. As long as the position (90° from lens to growing-axis of the plant organ) is constant, the absolute distance is of no importance. The final result of the analyzing process by “LambdaFit” is the parameter λ, which represents the state of the object’s form and is used for further analysis of form change over time.

### The Lambda Parameter

Since path curve geometry and in particular λ as the form-defining parameter play prominent roles in the present study design, we will very briefly and qualitatively describe the definition of λ. The mathematical background of λ and all necessary formula are explained in detail elsewhere (Derbidge et al., 2013).

**FIGURE 5:**
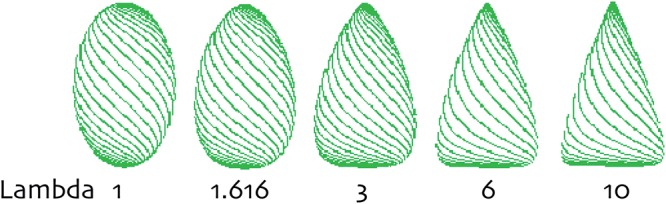
**The form-defining parameters Lambda (λ) of path curves**. λ > 1 yields egg- and cone-like shapes. The higher the λ-value, the sharper the shape is on top and the flatter it is at its bottom pole. Between 1 and 0, λ defines egg-like shapes that are flatter on the top and acuminated on the bottom. λ = 1 describes symmetrical shapes.

**FIGURE 6:**
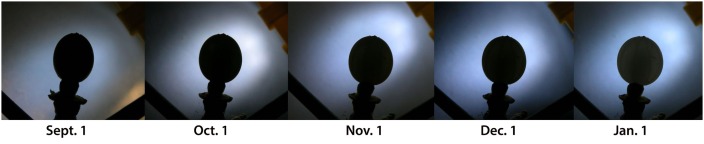
**The stages in change of form of a ripening mistletoe berry.** The λ -value of the profile changes from ≈ 1.2 in September to ≈ 0.9 in December. All pictures are of the same mistletoe berry (host tree oak) from 2013/14.

Since we are interested in the outline, we reduce the rather complicated mathematics to a two-dimensional system. An organ following a path curve (an egg-like shape) is a bi-symmetrical form with a mirror axis XY, with top (X) and bottom (Y) of the organ, which must be clearly defined, as in mistletoe berries. Every point on XY has two corresponding points (a and a’) 90° to the axis on both sides of the outline of the organ. Any two points a and a’ define a cross section through the organ at a certain level, which gives a diameter for this level. T = XY/2 cuts the line XY in half. For any chosen level, a λ_level_ can be defined as ratio of the chosen level diameter to the T level diameter. The parameter λ of the whole path curve then is defined as the ratio of the mean of all λ_level_ on XT to mean of all λ_level_ on YT. The parameter λ thus is an approach to describe the ratio of the curvature of the upper half to the curvature of the lower half of the objects outline shape. In other words, λ is a measure indicating the flatness or sharpness of an egg-like shaped form. λ = 1 thus describes outline shapes that are equally rounded on the top and bottom. λ-values > 1 describes egg forms that are more acuminated on the top and flatter on the bottom. λ < 1 but > 0 will be the opposite, flat on the top and peaked on the bottom. λ = 0 is the point of transformation to a new set of path-curve forms: for λ < 0 the path curves turn into vortex-like forms (see **Figure 5** for a range of λ shapes relevant to mistletoe berries). Mistletoe berries change their shape during ripening which λ slowly decreasing from λ ≈1.2 in September to ≈0.9 in December (see **Figure 6**). Their overall shape change, so to speak, is the inversion of an egg-like shape “standing upright” (i.e., flat on the bottom part and sharp at the top end) which moves toward the opposite: flatter on top and sharper at the bottom, where the berry is attached to the stem (egg standing upside down).

### Data Sets and Lambda Values

The λ-values represent the state of form for a certain picture extracted from the pictures via the software we use (Derbidge et al., 2013). The λ-values we use are mean values of 10 independent measurements of the same photograph (except for 2010 where it is the mean value of only six independent measurements). These 10 (6) measurements, performed in randomized order within a set of several pictures, allow calculation of a mean value and a corresponding standard deviation (Derbidge et al., 2013). This mean value is used in all further calculations.

To assess circadian rhythms, one photograph per hour H1, H2 … H24 was taken. Thus, 1 day yields 24 λ_j_ data points (j = 1, 2 … 24). In order to calculate mean circadian rhythms over several days, data from each day was normalized to the experimental mean of the corresponding day. A circadian data set for a given period of time (day 1–i) was calculated by averaging the normalized λj data for each hour j of the 24 data points for all the experimental days of the given period with the corresponding standard deviation (SD) or standard error (SE).

### Environmental Data

Weather data were obtained from the weather station run by “Meteo Swiss” in Binningen (BIN), Basel, Switzerland, situated approximately 7 km northwest from our location at a similar altitude (316 m above sea level). In addition, the temperature was recorded in the actual place where the berries were growing and compared with the temperature from BIN. No irregularities between the two measurements were found, except that range of temperature in the mistletoe huts was proportionally higher on warm sunny days (greenhouse effect). The site of observation is geomorphologically and meteorologically comparable with that of the weather station. Basically there are three meteorologically relevant weather situations: a weather front coming from the west; an inversion with fog; a stable high pressure (good weather) situation. All three will be the same in both places. No meteorologically relevant barriers separate both places. For the statistical correlation analysis we used the BIN data exclusively, since they are representative of our location and are recorded at the same time as the other weather data from BIN. All data from BIN are means or sums of the previous hour (see **Table 1**).

**Table 1:** List of weather factors recorded at the BIN weather station for the correlation analysis with λ.

Weather factor	Unit	Definition
Temperature (2 m above ground)	°C or K	Mean of previous hour
Air humidity	%	Mean of previous hour
Sunshine	min	Sum of previous hour
Global radiation	W/m^2^	Mean of previous hour
Atmospheric pressure	hPa	Mean of previous hour

*For some calculations we transferred temperature (°C) into K to avoid calculations with minus degree.*

### Deviation between Path Curve Form (Lambda) and Shape of Plant Organ as Represented in the Energy Value

The “energy” value, used in the software’s algorithms, is an operation analogous to the standard deviation and gives the average distance between the path curve and the berry’s contour. For each pixel t (0 ≤ t ≤ n) of the path curve, the distance to the respectively closest pixel of the plant contour dist_t_ is measured. The squares of all dist_t_ are summed up and divided by n, whereupon the square root is extracted. The formula is Equation 1:

(1) $$e=\sqrt{\frac{\Sigma_{t=1}^{n}{dist}_{t}^{2}}{n}}$$

This “energy” value *e* estimates the agreement between the actual contour of the mistletoe berry and the mathematical path curve fitted to that shape. Further details on the formula and the operations done are given elsewhere (Derbidge et al., 2013).

Notice that the pixel distance and hence the “energy” value depends on the picture’s resolution. For instance, if the height of a berry is 500 pixels and e is 3 pixels, there is a 0.6% deviation. For a real berry’s height of 10 mm the average deviation between berry outline and path curve is 0.06 mm.

### Statistical Analysis

Basic data reduction (for the operations mentioned see Data Sets and Lambda Values) were performed using Excel 12.3.2 (Microsoft, Redmond, WA, USA) data sheets. Data smoothing was done with LOWESS (locally weighted regression scatter plot smoothing) fits (Chambers et al., 1983), calculated with KaleidaGraph 4.0 (Synergy Software, Reading, PA, USA). This operation defines for each data point a linear regression equation, in which a variable percentage P of neighboring data points can be included. The function thus calculates a smoothed curve; the larger the percentage P of included neighboring data points, the smoother and straighter the curve. Correlation analyses were done using the Pearson product moment correlation test, calculated with Statistica 6.0 (StatSoft, Tulsa, OK, USA).

## Results

### Outline Shape of Mistletoe Berries Show a Correlation with Path Curves

**FIGURE 7:**
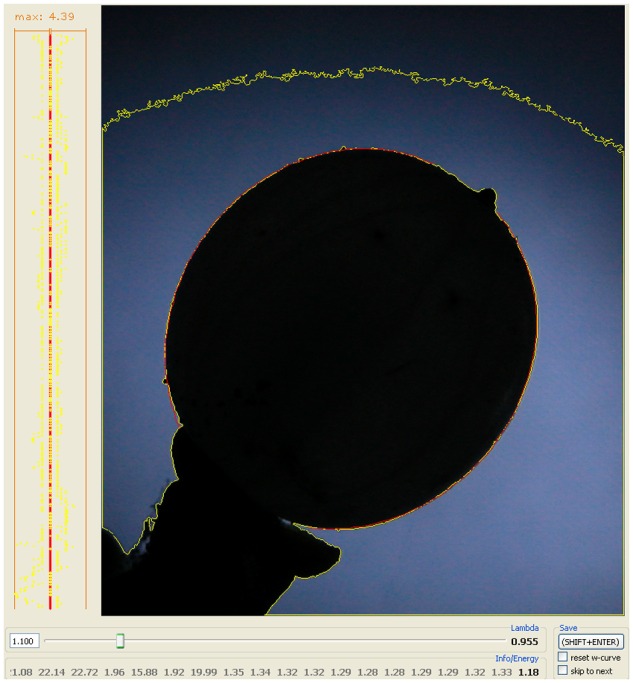
**Congruency between the mathematical (path curve, red) and the actual form (outline shape, yellow) of a mistletoe berry.** The yellow line is the outline of the black object determined by the LambdaFit software [λ] and the red line is the best fitting mathematical path curve to that given shape. On the left-hand frame the path curve is set as a straight line from top to bottom, and the deviation to the plant organ’s contour is shown for every pixel on the line for both sides. The number above that graph gives the maximum deviation. On some parts of the graph the red and the yellow line overlap, i.e., the path curve exactly fits the berry’s outline. The bottom part and the tip of the path curve are “cut off”, since the stem and the top (where the wilted petals are) must be excluded.

Mistletoe berries show an almost perfect congruence with mathematical path curves. The “energy” value, as implemented in the algorithms of the software used to optimize the fit of the path curves to the outline shape of the mistletoe berries, is closely related to the standard deviation of the distance between fitted curve and outline (see Methods for details). The “energy” value depends on the picture’s resolution, since it is calculated based on the deviation in pixels. The average “energy” value is between 0.9 and 1.5 pixels (see **Table 2** for the average λ and energy values of all the circadian rhythm photo-series used in this study), which corresponds to an average “deviation” between fitted path curve and berry outline shape of 1.6–2.7 μm for the average height of a berry of 9 mm. Thus, the outline of mistletoe berries corresponded almost perfectly to mathematical path curves for all four measurement series between 2010 and 2013 (for a visual impression see **Figure 7**).

**Table 2:** Average λ and energy values (mean ± standard error SE) for four series of measurements in 4 years.

Type of measurement series	Average λ (mean ±*SE*)	Average energy value [pixel] (mean ±*SE*)
Mistletoe berry 2010 (lilac); *n* Pictures = 380; *n* Measurements = 2280	1.1309 ± 0.0165	1.5022 ± 0.1008
Mistletoe berry 2011 (oak); *n* Pictures = 144; *n* Measurements = 1440	1.0553 ± 0.0320	0.9740 ± 0.1140
Mistletoe berry 2012 (oak); *n* Pictures = 144; *n* Measurements = 1440	0.9823 ± 0.0299	1.2844 ± 0.1651
Mistletoe berry 2013 (oak); *n* Pictures = 144; *n* Measurements = 1440	1.0804 ± 0.0146	0.8797 ± 0.0605

*λ is the form-determining parameter of the path curve, and the energy value corresponds to the deviation between the mathematical path curve and the outline shape of the mistletoe berry (expressed in pixels). 144 photographs (380 in the year 2010) of a mistletoe berry were taken during 6 days (16 days in the year 2010, with 4 single photos missing due to technical problems) at hourly intervals. λ was measured in randomized order within a given experimental series (n = 10 independent measurements per photo (n = 6 in the year 2010)).*

### Circadian Rhythm

**FIGURE 8:**
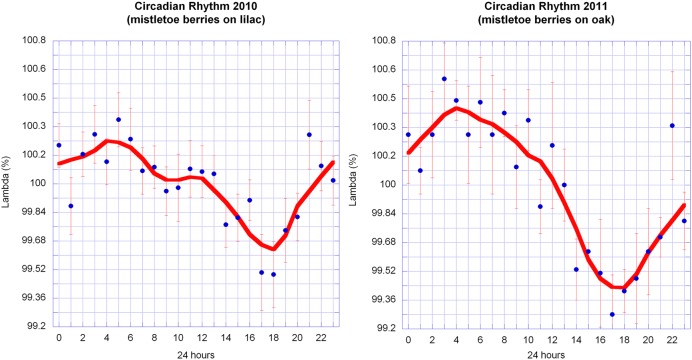
**Circadian rhythms of λ in mistletoe berries in October 2010 and 2011.** Hourly data from 16 days in 2010 and 6 days in 2011 were normalized for each day and averaged (mean ± SE). The graphs of both years share similar features: the maxima are at about 4–5 a.m. and the minima are at about 5–6 p.m. The smooth continuous lines are LOWESS fits (*p* = 30%).

Once it was clear that the outline shape of mistletoe berries could be mapped as path curves, four series of pictures from the years 2010–2013 were assessed in order to identify potential circadian rhythms. Each series consisted of 6 days in October of uninterrupted data (16 days in 2010). Statistical analysis revealed highly significant circadian rhythms in 2010 and 2011, and no significant rhythms in 2012 and 2013 (**Table 3**; **Figure 8**). A correlation analysis of the circadian data from 2010 to 2011 revealed a highly significant correlation between circadian rhythms from both years (*r* = 0.7829; *p* = < 0.0001; *n* = 24). The daily rhythms of 2010 and 2011 are in congruence. Compared to the data of 2010/2011, the standard deviations of the hourly mean values of the 2012/2013 daily data are much higher, an indication that there is no daily rhythm or signal in those 2 years (see **Table 3**).

**Table 3:** Results of the ANOVA analysis (24 groups) investigating possible circadian rhythms (data of **Figure 2** and respective measurements for the stated years).

Year	Month	Host-tree	Groups	*F*	*p*
2010	Oct	Lilac	0–23 (each hour) over 16 days	2.269	0.001
2011	Oct	Oak	0–23 (each hour) over 6 days	5.498	<0.001
2012	Oct	Oak	0–23 (each hour) over 6 days	1.233	0.231
2013	Oct	Oak	0–23 (each hour) over 6 days	0.117	1.000

*F: F-test values; p: significance levels.*

### Determining Weather Influence

To search for possible causes for the circadian rhythms in the berries’ change of outline shape in the years 2010 and 2011, a number of correlation analyses were performed with factors known to have an impact on circadian rhythms (Dunlap et al., 2004; Yakir et al., 2007; Pfeuty et al., 2012). The weather data of the days with λ data were normalized for each day and averaged. Correlations between all weather factors with λ were calculated (**Table 4**).

**Table 4:** Pearson correlation coefficients r and corresponding significance levels *p* between circadian rhythms (λ) and the different weather factors.

Correlations Lambda (λ) with weather factors	2010 *N* = 380		2011 *N* = 144		2012 *N* = 144		2013 *N* = 144	
	*r*	*p*	*r*	*p*	*r*	*p*	*r*	*p*
Temperature	-0.2098	**<0.0001**	-0.5737	**<0.0001**	0.0418	0.6185	0.0865	0.3042
Air humidity	0.2528	**<0.0001**	0.6226	**<0.0001**	0.0269	0.7485	-0.1006	0.2318
Sunshine	-0.0987	0.0547	-0.3940	**<0.0001**	0.1489	0.0749	-0.1207	0.1511
Global radiation	-0.0655	0.2026	-0.3243	**0.0001**	0.1034	0.2175	-0.1088	0.1959
Atmospheric pressure	-0.0258	0.6165	0.1216	0.1466	-0.0093	0.9122	-0.4703	**<0.0001**

*Significant values (p < 0.05) are printed in bold*.

**FIGURE 9:**
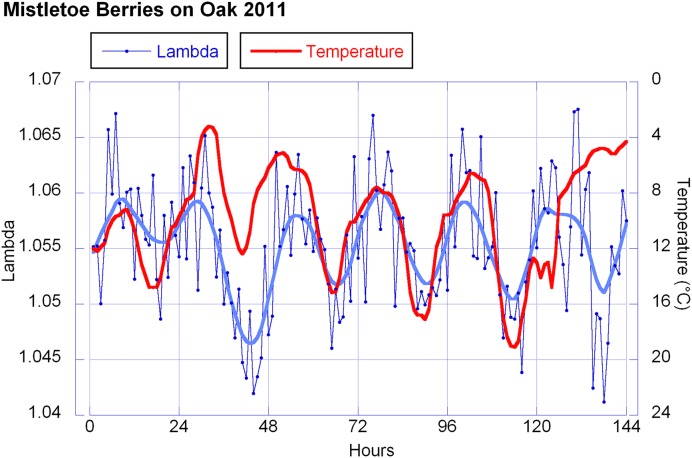
**Lambda values and temperature.** Hourly λ-values in blue (6 days = 144 h) and temperature values in red (reverse axis). For λ every data point represents the mean of 10 measurements. For the temperature each dot represents the average of that hour. The smooth continuous line represents a LOWESS fit for the λ data with *p* = 10%.

**FIGURE 10:**
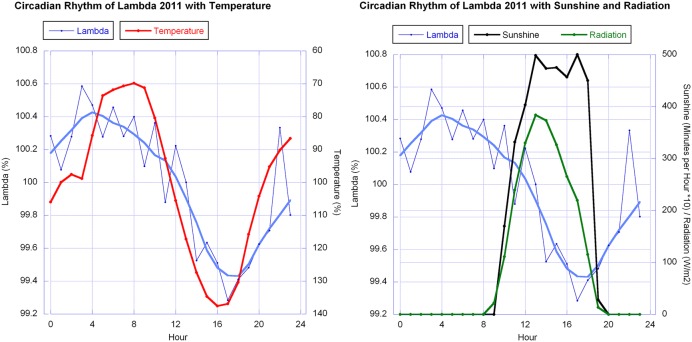
**Circadian rhythms of λ in mistletoe berries (error bars omitted for clarity) in October 2011 together with, left: the daily change in temperature (reverse axis), and right: sunshine minutes and global radiation.** The smooth continuous lines for λ (blue thick line) are LOWESS fits (*p* = 30%). λ and temperature data were normalized for each day and averaged. They are expressed as relative percentage change whereby sunshine [minutes/hour] and global radiation [W/m^2^] are the average of the 6 days in regard to the specific hour.

Temperature and humidity (humidity in reciprocal relationship with temperature) show highly significant correlations with the berries’ outline form (λ). This corresponds to wave patterns similar to the λ wave when viewed in a graphic representation for 2010 and 2011 (see **Figure 9** for 2011). Sunshine hours and global radiation show a significant correlation in the 2011 data set (see **Figure 10**).

### External Factors for a Strong Diurnal Movement

The fact that we found significant circadian rhythmic change in mistletoe berries in 2010 and 2011 but not in 2012 and 2013 (see above and **Table 3**) raised questions that we investigated by looking at weather conditions that might trigger or suppress the rhythm.

**FIGURE 11:**
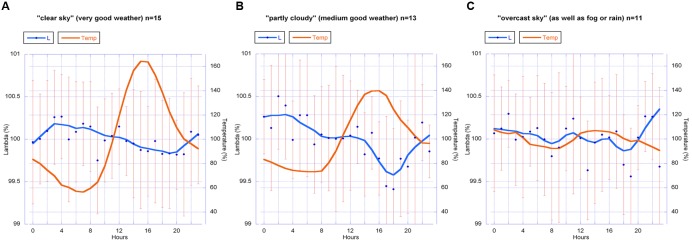
**Circadian rhythm (blue line) of all λ data (2010–2013) for the groups (A)** “clear sky,” **(B)** “partly cloudy” and **(C)** “overcast sky,” with the daily change in temperature (red line). λ-values are mean values with standard deviation (SD). Graph b shows the most distinct circadian rhythm, similar to the circadian rhythm found in the data from 2010 and 2011 (**Figure 8**). All data were normalized for each day and averaged. Group size (24 h sets analog to **Figures 8–10**): *n* = 15 **(A)**, *n* = 13 **(B)**, *n* = 11 **(C)**. The smooth continuous line is a LOWESS fit of the λ data (*p* = 30%).

Therefore all data were further analyzed. A histogram of all 24 h data blocks with regard to sunshine minutes per day showed a distribution with three distinct groups. We separated the 24 h data blocks of all years into these three groups as follows: (a) “clear sky,” i.e., good weather with a high number of sunshine minutes (>400 min per day); (b) “partly cloudy,” i.e., moderately good weather (with sunshine minutes between 100 and 400 per day); and (c) “overcast sky” i.e., bad weather (<100 sunshine minutes per day or no direct sun at all). Those “bad weather days” were rainy, foggy or overcast because of weather changes. This grouping corresponds to the range or amplitude of temperature (high amplitude = no clouds; medium amplitude = partly clouded days; and low amplitude = clouds or fog or mist; see **Figure 11**).

The graphs of the circadian rhythms of λ show: a significant change of form for group (b) (“partly cloudy”) in a rhythm that accords with **Figure 10** for 2010 and 2011; a visually clear but statistically just near significant change (*p* = 0.06) of form in group (a) (“clear sky”); and no pronounced change in λ in group (c) (“overcast sky”).

Analysis of variance tests showed that an intermediate weather situation with medium amplitude of temperature and a medium number of sunshine minutes per day are the necessary condition for the strongest rhythmic change in λ. This result indicates that the change of form is sensitive and cannot be fully expressed by the berries when sunshine is strong (i.e., high global radiation [W/m^2^]) and also when the sky is under full cloud cover. As these are indirect assumptions, the results elicit certain questions rather than answering them. But a hypothesis that change of form is in this case dependent on “nice weather” (a not too extreme temperature amplitude, and also a not fully overcast sky) seems reasonable. Some parts of the sky must be blue (but no direct sun, at least not all the time) for the emergence of this change of form as a statistically distinct feature (see **Table 5**).

**Table 5:** Analysis of variance (ANOVA) results for the circadian rhythm analysis of form change as a function of three characteristic weather groups.

Group	Analysis of variance		Weather features	
	*F*	*p*	Temperature Amplitude	Sunshine minutes (total per day)
(a) “clear sky”	2.219	0.0665	very high	>400
(b) “partly cloudy”	3.636	0.0065	pronounced but intermediate	100–400
(c) “overcast sky”	0.459	0.8063	small	<100

*Group size: a (n = 15), b (n = 13), and c (n = 11). The data correspond to **Figure 11**. The group b (“partly cloudy”) with medium amplitude in temperature yields the most pronounced result for circadian rhythms.*

## Discussion

### Research Design and Methodology

We measured circadian morphological changes affecting the outer skin of a berry, something that has not been previously studied to the best of our knowledge. The only research with similarity to our approach is on diurnal changes in leaf hyponasty or growing rates, studies that are summed up under the topic of plant movements (Klein, 2007; Asanidze et al., 2011; Dornbusch et al., 2012; Chitwood, 2014). The rationale for those studies is, largely, to describe phenological findings and relate them to physiological and molecular processes and structures within the plants. But additionally phenological change detection from pictures is used with modern techniques to, for instance, trace growth (Li and Gu, 2004). The focus on exact form change in berry phenology in our approach *in vivo* and *in situ* using mathematics from projective geometry is new. The rhythms found are phenological changes in outline shape that are strictly mathematical and yet dynamic in nature (path curves). The outline as well as the detected outline variance can be accurately mapped with the λ parameter. λ thus becomes the key value to trace the change of shape. One formula can trace changes in form because only one parameter (λ) is changing its value. Since our focus is change of form, we cannot say anything about anatomical or physiological reasons for the changes, or corresponding metabolism within the berries. We strictly analyze the correlation between a mathematical law and a biological object to demonstrate their correlation in this study.

### Findings Relating to the Path Curves of Mistletoe Berries

To the best of our knowledge, forms or outline shapes have not yet been used in plant biology or in any research involving living organs in the manner we applied them in our study. In projective geometry, path curves are a set of dynamic forms with determining parameters accessible to exact measurement (Edwards, 2003; Klein, 2006; Derbidge et al., 2013; Klein, 2013). This new method of shape recognition using mathematics derived from projective geometry works under different outdoor conditions (like day and night, change in temperature, change of relative humidity) and detects a difference of just a few pixels between formula and real object (see Results, **Table 2**; how to convert pixels into actual berry size, which is depended on the pictures resolution, is exemplified in Derbidge et al., 2013).

Form as such is used to describe objects at all levels, i.e., from whole organisms, through organs to small units such as cells, or even molecular structures that can be reconstructed as two- or three- dimensional forms (Thompson, 1992; Pivar, 2009; Schwartz, 2013). Forms of plant organs such as leaf forms have been classified and are used to determine species but rarely to detect circadian or other rhythms.

Using λ in the algorithm to define the state of the outline shape of mistletoe berries might open a new line of thought in chronobiology. This feature, visible to the eye, makes studying the phenomena accessible without the aid of complex and expensive apparatus. The method described in this publication thus is an *in vivo* and *in situ* observation during the long ripening season of mistletoe berries.

The approach we seek to introduce here, if proven to be of value, could re-engender something of a more direct, phenotypic relationship to nature while at the same time continuing to pursue current biological questions such as those of rhythmic interactions or behavior.

Form, in its common usage, is about static features: proportions, length, width, number of features, etc. (Lonergran, 1985; Ji-xiang et al., 2013; Schwartz, 2013). Path curves, by contrast, are dynamic in nature (see **Figure 7**). There was a significant correlation between a path curve’s outline shape and that of mistletoe berries. Here, we demonstrated that it is promising to pursue this approach further and to extend it to chronobiology.

### Findings Relating to the Circadian Rhythm

It was not completely unexpected that mistletoe berries exhibit a circadian rhythm since almost all living organs so far studied do so (Kondo and Ishiura, 1999). Almost, all biological functions find an expression in rhythms. When plotted in graphs, the circadian rhythm of the berry’s outline shape (change of λ over time) appears to be quite close to a harmonious wave, i.e., a sine wave without any disruptive features such as asymmetry or certain peaks. Thus, it expresses a common rhythmic behavior found in many organs or organisms (Kondo and Ishiura, 1999; Dunlap et al., 2004).

Circadian rhythms are well-understood by means of circadian clocks – genetic oscillators that generate biochemical rhythms with a free-running period (FRP) close to 24 h (Roenneberg and Foster, 1997; Somer et al., 1998; Dunlap, 1999; Young and Kay, 2001; Bell-Pedersen et al., 2005; Pfeuty et al., 2012). To achieve synchronization with the day/night cycle, a circadian oscillator integrates a complex set of environmental signals such as changes in daylight quantity or quality (Young and Kay, 2001; Bell-Pedersen et al., 2005) or in temperature (Yoshii et al., 2009). The effect of those signals usually alters the reactions, i.e., the characteristics of the rhythm. In particular, light signaling pathways play a major role in coupling the clock to its environment as they provide information about the day–night status.

### Findings Relating to Weather Influences

The observation of the mistletoe berries and the detection of their change of form in four consecutive years took place under different specific weather conditions. All weather data were taken from the nearest official and validated weather station (7 km distance from our study site). We correlated those weather data (temperature from BIN) with local data (temperature measured in the hut around the observed berry). Correlation analysis showed that both weather situations were highly significant in agreement. So we decided to use the more reliable “official” data from a state weather station. The host tree’s leaves shaded the study site, so no direct sunlight reached the hut construction that allows good air circulation and statistically, the rather small greenhouse effects could be neglected. Humidity always correlates reciprocally with temperature and all other weather factors we used do not differ locally from the nearby weather station, which, meteorologically, is in a very comparable location.

A certain circadian rhythm of the mistletoe berry’s shape was observed in 2010 and 2011, but not in 2012 and 2013. Correlations to λ were observed in the data of 2010 and 2011 for temperature and humidity, and in 2011 also for sunshine hours and global radiation (graphs of these data of all 4 years are available as Supplementary Figure A).

The λ correlations in 2010 and 2011 with temperature and sunshine minutes or radiation don’t match ideally. λ and temperature both show a wave, close to a sine wave in its rise and fall, with almost equidistant periods. Thus both waves have a similar sine curve, but with a phase shift. The λ wave has a maximum at about 5 a.m., 2 h before sunrise (which is around 7 a.m. in October at the place of observation). The temperature drops to its minimum a bit later (minimum at 8 a.m. which is a 3 h shift from the λ wave). In the afternoon the λ wave minimum is at 5 p.m., 1 h later than the temperature maximum but running earlier than the decline of radiation (sunset at 7 p.m.).

Temperature and light influences are fundamental causes of circadian rhythms but the rhythmical change of form in the present case do not correspond to these factors in an unambiguous way.

In plants it seems to be usual for effects to follow the timekeeper and not the other way around (Roenneberg and Foster, 1997; Beersma et al., 1999; Pfeuty et al., 2012).

An exploratory analysis identified predominantly blue vs. overcast sky as being correlated to the presence of a circadian phenotypic shape change. During “nice” weather the diurnal λ rhythm was observed, but not in “bad” weather. Most of the 2012 and 2013 weather was foggy or the sky was overcast at the time of measurements, which seemed to suppress the rhythmic expression in λ change.

### The Influence of Light

In our research the “usual suspect,” light, as major influence on circadian rhythms, has not been exhaustively studied yet. For instance, it would be necessary to further study the influence of day length. Since we studied rhythm in a seasonal context the impact of the light situation (for instance total sunshine minutes per day) on the characteristic circadian pattern found, could be of significance. Especially, in October and November when the duration of daylight changes rapidly (in Middle Europe). The time of minima and maxima might therefore vary from the beginning to the end of the measurement period: in September, for instance, there is at least one additional hour of light compared with November. We measured berries only once per hour (24 measurements per day), and therefore did not obtain a high enough resolution for change in day length to resolve this question. Measurements would be needed every 10 min or less to detect possible (and most likely small) changes during the twilight hours.

Knowledge of circadian rhythms is extensive and sophisticated. Modifications to rhythms during the season as an adaptation to changes in habitat conditions, or during the day, for example when overcast sky situations can rapidly alter the quality and quantity of light, have been discussed for different organisms (Beersma et al., 1999; Dunlap, 1999; Pfeuty et al., 2012).

The mistletoe berry has no testa, being a pseudo-fruit and bearing a pseudo-seed with mostly one, but sometimes 2 and rarely 3 to 5 embryos, each with a haustorium that emerges from the endosperm, a hypocotyl and two rudimentary cotyledons. As such it is probably sensitive to alterations in light quantity and quality, since the new organism growing within the ripening berry is already active in photosynthesis (Becker, 1986). Our discovery that full sun exposure flattens the peak shape of the 24 h-wave, contradicts an explanation of the rhythm as an adaptation to photosynthesis of the embryo. Half-clouded skies – conditions where the sun is not at full strength – seem to be optimum for expression of the circadian rhythm. These conditions are comparable with conditions in the morning and evening, when the sun is lower, but light is present.

Daylight is the key factor for circadian clock entrainment. But it changes in quality and quantity seasonally and during the day due to changes in weather such as cloud-cover, and is therefore neither a reliable nor a uniform environmental cue (Beersma et al., 1999; Troein et al., 2011). Plants and other organisms need to be adapted to such changes (Stoleru et al., 2004; Trivedi et al., 2013). It is likely that a physiological study of mistletoe berries will discover the effect of these influences (Büssing, 2000; Urech et al., 2006; Dorka et al., 2007), but our findings tend to show that the outline form is not clearly dependent on such factors. Light, by means of direct sunlight and radiation is linked to temperature, a mayor possible influence on form change as discussed in Section “Findings Relating to Weather Influences.”

### Mistletoe Physiology and Anatomy

The results presented here should be contextualized with the berries’ anatomy and physiology, which we refer to collectively as “internal” biotic factors. Anatomical structure and physiological activities of the mistletoe berry are of great interest, since the changes of shape must be accompanied by anatomical rearrangements and alterations in physiological processes (Azuma et al., 2000; Dorka et al., 2007).

Sap flow has been studied as a rhythmic circadian and seasonal phenomenon especially in regard to the metabolism of plants, which is subject to external influences (Zürcher et al., 1998; Zürcher, 2001; Barbeta et al., 2012; Forster, 2014). Sap flow has been studied in many species including various oak subspecies (Ćermák et al., 1982; Granier et al., 1994; Barbeta et al., 2012). Recently the topic gained attention in regard to nocturnal sap flow (Dodd et al., 2005; Pfautsch et al., 2011; Forster, 2014). In general the strong increase in xylem sap rise from roots to leaves starts at sunrise (when light meets the leaves and photosynthesis starts). In the broadly recognized cohesion-tension theory, the factors responsible for this mechanism are driven by transpiration, i.e., the sap flow rhythm is correlated with sunlight and modified by temperature as well as humidity in the environment. On a hot day or during a dry hot summer (with light), as for example in Mediterranean countries, water flow is reduced due to stomata aperture regulation to avoid dehydration (Pfautsch et al., 2011; Barbeta et al., 2012). But even under these conditions the diurnal change in water volume rising in the tree starts at sunrise and rapidly decreases at dusk (Pfautsch et al., 2011). This is a rhythm quite different from the one we found in the change of form in mistletoe berries. **Figure 4** shows the sine like rhythm of change of form of the berries’ outline shape together with sunlight (sunshine minutes per hour) and global radiation. According to the literature on sap flow, these two parameters should quite accurately correlate with sap flow, but they don’t correlate well with the change of form. Thus tree xylem flow most probably is not the cause for the rhythmic form change observed. The rhythm of the tides, i.e., the moon’s influence, has also been subject of some studies and would be worth looking at, especially since it has generated much controversial discussion (Zürcher et al., 1998; Vesala et al., 2000; Zürcher, 2001; Barlow et al., 2010). Causes of any detected rhythms in sap flow would demand a similar or even synchronous study of sap flow in the host tree and the mistletoe growing on it. To our knowledge no such study yet exists and it would be a good candidate for further research where the interaction of host tree and mistletoe (mutual or parasitic relationship) could be the focus.

Little is known about transpiration of mistletoe berries. They seem to have a cuticle that effectively prevents evapotranspiration (Sallé, 1983; Büssing, 2000). Still, in principle, it could be possible that sap or water flow from the host tree into the mistletoe bush (stem and leaves) also affects the mistletoe berry even though the latter most probably doesn’t need water for transpiration. However, as discussed above, tree sap flow cannot be the only cause of the change of form of the mistletoe berries.

**FIGURE 12:**
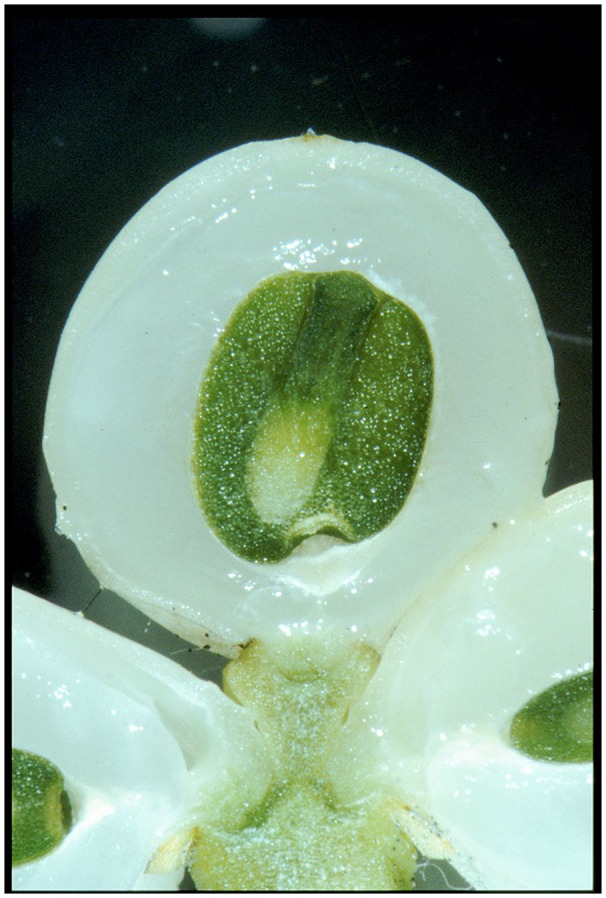
**Longitudinal section of a ripe mistletoe berry in November.** The green embryo with its clearly visible hypocotyl and surrounding endosperm is encircled by the white pericarp. The otherwise strong tension of the skin has collapsed because of the cutting needed for the section (Photo: H. Ramm).

A mistletoe berry exhibits specific anatomical features. The green embryo and endosperm lie in the berry’s center and are surrounded by the pericarp. The mesocarp develops as the main substance of the berry – a whitish, transparent, soft, and glue-like material. The mesocarp is composed of inner elongated cells and outer vacuolated cells. The elastic and flexible skin (epicarp) has a thick cuticle (Sallé, 1983) (see **Figure 12**). These anatomical features, in principle, should enable the occurrence of a dynamic form change. Further detailed investigations of the underlying changes in the anatomical structures and the physiological processes are needed to provide a rationale for the specific shape changes observed.

The germination of a mistletoe embryo starts by end of September. The hypocotyl then begins to grow toward the epicarp. In cross sections one can see that it breaks through the endocarp and, after January, changes the outline shape of the berry (its skin) since it pushes from inside against it. In December the hypocotyls (there can be one, in some cases two or even 3–5 embryos per berry) can be clearly seen through the transparent skin. Unpublished measurements about the weight of the berries show a steady increase of weight until the end of December (Urech, personal communication). This probably is due to the embryos’ growth. Whether this weight gain is paralleled by a growth in size of the whole berry, cannot be stated (λ is a dimensionless ratio). Increase in weight could also be explained by an increase in density. But this result suggests that the mistletoe berries we observed in October were still in a ripening phase. If the change of form has to do with the ripening process, it might be a rhythmical growing process or be induced by the physiological activity of the embryo.

### Methods

Within the present research project a new method for studying rhythms on a macro-phenotypic scale was developed: the detection of rhythmic changes in living objects *in situ* by controlled outdoor photography and mathematical processing to fit the projective geometrical forms. In a former publication (Derbidge et al., 2013) we reported on the algorithms and software used for this study. The present paper focuses on the handling of mistletoe, the photography and the equipment used. Overall the results demonstrate the robustness and reliability of the method itself. Questions and problems of the newly developed method will be identified and discussed below.

The procedure used allows photography to be undertaken in a situation as natural as possible, and guarantees the comparability and uniformity of picture series, i.e., equal lighting of pictures taken in daylight and in artificial lighting at night, for purposes of comparison. Most importantly, it minimizes the variation of angle and distance between lens and object.

The outdoor situation sometimes results in unpredictable elements. Extreme weather situations such as storms or a sudden drop of temperature can bias the data or lead to missing data due to damage to the plant. For example, a very sudden drop in temperature, associated with a rapid increase in relative air humidity, can fog the lens. Storms can make the tree shake so much that the fixed stem with the berry can change position or even break; and identical repositioning is not possible. In addition, natural “hazards” can occur, such as the berries being eaten by birds. Flies and other insects were seen on some pictures, making them useless for measurements. Fungi and insects find favorable conditions in the sheltered situation during summertime, which means that the roof and side protection of the hut have to be removed in summer.

The cameras and the other outside equipment need to function in a temperature range from -15 to +35°C. This is quite a technical challenge. Thus the status quo described is a compromise, yet one that meets our needs and proved successful.

The hut construction leads to very small, but measurable greenhouse effects on warm, sunny days. A possible influence could not be estimated in the present experimental setting; a closed, air-conditioned system would be necessary for comparison.

Certain other influences could not or cannot easily be detected. For instance, do the LED flashlights (Jeong et al., 2012) or the electromagnetic fields around the berries (arising from the camera, the light and the “trigger-finger”) influence or distort the biological object? And if so, how severe is the influence and/or is it negligible?

To reduce the influence of the artificial lighting, it is set to turn on each hour for only 3 s with a smooth run-in and fade-out phase. Nevertheless it is not possible to eliminate the possibility of light influence since light is regarded as the most important trigger in chronobiology (Young and Kay, 2001; Dunlap et al., 2004).

The need to obtain optimum photographic contours of the berries with negligible biological effects necessitated a long sequence of experiments, and some aspects could still be improved. However, the methods used are well-suited to the task and yielded robust results. As shown elsewhere (Derbidge et al., 2013) and confirmed here, a compromise between external observation and repeatable and comparable results is possible.

### Research Questions Raised by the Results

The demonstrated fact, that the change in profile form of a biological organ (a mistletoe berry) can be accurately described with a particular mathematical form (a path curve from projective geometry), and about using path curves in describing the dynamics of form in phenotype, invite questions about the link between form and biology. The phenomena observed could open up new aspects in this field, which is a highly topical one in current biology (Müller and Newman, 2003; Dornbusch et al., 2012; Newman and Linde-Medina, 2013). A next step could be to enquire further into the physiological origin of the change of form.

Many scientific and laymen authors have been fascinated by the occurrence of mathematical form in biology (Arber, 1950; Kappraff, 2002), yet they rarely explain scientifically how that correlation comes about. In some cases, such as golden section geometry in the seed order of a sunflower, the phenomenon can be explained by means of optimum space use as a useful adaptation. Yet the precise adaptation values of those forms or patterns has not, or cannot be explained, because many other options would lead to similar success (Müller and Newman, 2003; Dornbusch et al., 2012; Newman and Linde-Medina, 2013). Ultimately the old questions (e.g., “what is form?”) remain, and we will probably continue to ponder them for as long as scientists seek to understand how form and structure arise in evolution in an ordered and purposeful way (West-Eberhard, 1989; Waldrop, 1992; Carroll, 2005; Kirschner and Gerhart, 2006; Holland, 2010).

### Future Research

The results presented here are pertinent and raise a wide range of questions for future studies. Ongoing research will continue to focus on rhythms and their correlations at a macro-phenotypic level. Further research is needed to explain and better understand the connection between form and function in the case of rhythmic change of shapes. As yet we do not know how general it is for plant organs to follow a mathematical or projective geometrical form. But clearly, “form follows function” is not a satisfying answer here, since there is no recognizable function prior to the phenomenon. If fruiting and ripening have a biological function alongside the obvious and well-studied function of seed production, further research may change how we look at plants in many ways. The empirical data introduced here need to be integrated into a theoretical context, which could open up a novel field of potential inquiries. One of the first steps would be, for instance, to examine other plants (fruits) to discover whether the phenomenon of rhythmic change of form in mistletoe is an exception or not. Likewise, it would be of great interest to study morphological change in different species to ascertain differences from or similarities to the phenomena identified here.

The results of the present study set a clear path to continue the investigation and extend its applications to different objects that follow path curves, such as other berries, buds, and to other research. As showed by Edwards (2006), the heartbeat can be also modeled with path curves. This may be true of other living organisms and processes that have not yet been explored in this way.

## Author Contributions

RD and SB conceived and designed the study, with suggestions from PH. RD performed the experiment set-up, collected the data and analyzed them. RD and SB did the statistics. RD wrote the main manuscript, and all authors reviewed the manuscript.

## Conflict of Interest Statement

The authors declare that the research was conducted in the absence of any commercial or financial relationships that could be construed as a potential conflict of interest.
