# Supplementary figures and images for: Mistletoe Berry Outline Mapping with a Path Curve Function and Recording the Circadian Rhythm of Their Phenotypic Shape Change
Source: Front Plant Sci. 2016 Nov 25;7:1749. doi: 10.3389/fpls.2016.01749 (PMC5122707; doi:10.3389/fpls.2016.01749)

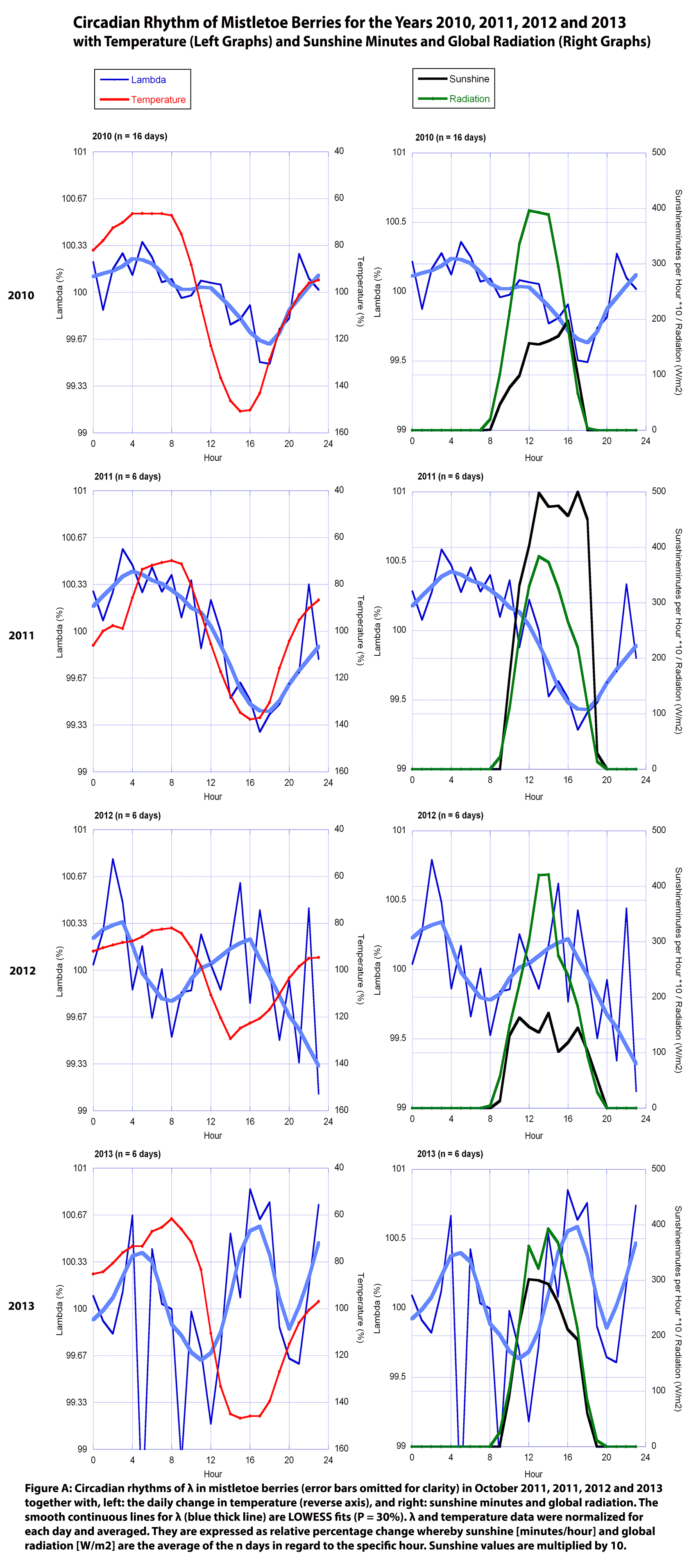

Supplement: Supplementary file 1 [file Image_1.TIF]
